# Innovations in Older Adult Music Andragogy: A Gerontological Framework for Adaptive Learning

**DOI:** 10.1093/geroni/igaf122.3403

**Published:** 2025-12-31

**Authors:** Bei-Yun Wang

**Affiliations:** National Chung Cheng University, Chiayi, Taiwan (Republic of China)

**Keywords:** Gerontology, Andragogy, Older Adult Music Education, Adaptive Learning, Public Spaces

## Abstract

This study employs an andragogical perspective and a gerontological framework to examine innovative approaches in older adult music education. Focusing on a music appreciation course at a community-based learning program for older adults, it investigates how personalized teaching strategies, diverse musical content (from classical works to film scores), and multi-sensory methods address older people’s cognitive and social needs. Based on qualitative data from one instructor and eight older learners (aged 63 to 77), the findings underscore the importance of linking life histories with new musical knowledge to enhance motivation, memory retention, and interpersonal engagement. Additionally, incorporating digital platforms and extending learning to public spaces, such as concert halls, fosters a more sustained impact on learners’ social interactions and quality of life. The study offers practical insights into designing adaptive andragogical practices, wherein older learners’ experiences serve as a vital foundation for curriculum development, thus contributing to broader gerontological discourse and advancing lifelong learning initiatives.

